# Quantifying the Effect of Intermonomer Improper Angles on Electron Delocalization in Conjugated Polymers

**DOI:** 10.1021/acs.jpcb.5c02849

**Published:** 2025-07-14

**Authors:** Robert S. Ramji, Andrew T. Kleinschmidt, Shruti Bhamidipati, Leon Zhang, Alexander X. Chen, Tod A. Pascal, Darren J. Lipomi

**Affiliations:** † Aiiso Yufeng Li Family Department of Chemical and Nano Engineering, 8784 UC San Diego, La Jolla, California 92093-0448, United States; ‡ NSF Materials Research Science and Engineering Center, 8784 UC San Diego, La Jolla, California 92093-0448, United States; § 428090 Schrödinger Inc, San Diego, California 92121, United States; ∥ Department of Applied Physics, Stanford University, Palo Alto, California 94305-6104, United States; ⊥ 124301 Lam Research, Fremont, California 94538-0000, United States; # Department of Chemical Engineering, University of Rochester, Rochester, New York 14627, United States

## Abstract

Electron delocalization between monomers in conjugated polymers influences both the charge transport and the mechanical properties of this class of materials. While this delocalization is generally understood to be maximized by coplanar monomer arrangements that enable π-orbital overlap, the precise geometric factors controlling delocalization remain incompletely characterized. Current molecular mechanics (MM) models of conjugated polymers primarily focus on intermonomer dihedral torsion, neglecting the potential effects of out-of-plane (“improper”) torsion between monomers. Here, we develop a method to isolate and quantify improper dihedral torsion effects on the electronic structure of dimer or donor–acceptor unit structures for three representative polymers: P3HT, PTB7, and PNDI-T. Quantum mechanical calculations reveal that improper torsion generally disrupts electronic delocalization, though PNDI-T maintains significant conjugation even at improper angles up to 30°—likely due to its extended π-system. While coupling between improper and dihedral torsion is observed, the energetic effects appear to be minimal (<1 kcal/mol) under typical conditions. These findings suggest that existing MM models may safely omit improper torsion parameters for simulations of local structures and motivates future studies aimed at quantifying its effect on larger-scale polymer films. Finally, to aid interpretation of these results, we have developed an interactive visualization tool that simultaneously displays delocalization energy profiles and 3D electron density isosurfaces.

## Graphical abstract

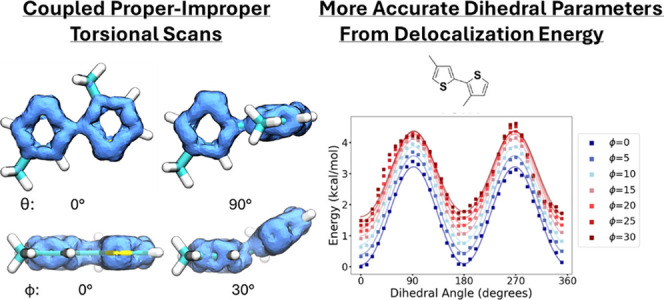

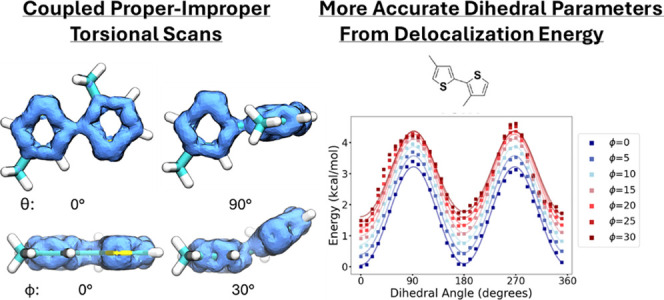

## Introduction

π-Conjugated polymers are characterized by a motif of alternating double and single bonds along the backbone. In this arrangement, electrons may delocalize from the bonding orbitals from which they originate. This delocalization of electrons along the backbones of conjugated polymers gives rise to an optical bandgap and semiconducting behavior.^1–3^ Delocalization is energetically favorable and usually depends on the degree of alignment between the π-bonds in the backbone, with notable exceptions for certain monomer selections.^4^ A planar conformation of sequential bonds maximizes this overlap, while deviations from planarity reduce it. Thus, electronic delocalization acts as a driving force toward the coplanar configuration of monomers.^5^ As a consequence of this drive toward coplanarity, conjugated polymers tend to be more rigid than saturated polymers.^6,7^ This relationship between electronic structure, optoelectronic properties, and the mechanical flexibility of conjugated polymers has motivated a number of studies focusing on understanding and accurately modeling intermonomer rigidity in conjugated systems.^8,9^

Part of the motivation for computational studies is that the backbone planarity of a conjugated polymer is difficult to measure experimentally.^10^ In these studies, it is typical to compute the energy as a function of the proper dihedral torsional angle between monomers (Figure 1b). Hereon, the term “dihedral torsion” will be used to refer to *intermonomer proper dihedral torsion*, i.e., a ‘twisting’ angle between two conjugated monomers about the connecting bond. Dihedral torsion can be likened to the spinning of a revolving door, where the degree of rotation around the central axis corresponds to the torsion angle between atoms in the molecule. However, deviation from planarity between monomers can also occur through improper dihedral torsions (Figure 1c). These *intermonomer improper dihedral torsion* occurs when the motion of the polymer produces an out-of-plane ‘bending’ between monomers. This motion occurs in the direction of the normal vector relative to the orientation of the conjugated ring. Improper torsion can be likened to the motion of a door hinge, with the center of mass of one monomer moving as the torsional angle increases (Figure 1). In this work, we will simply refer to *intermonomer improper dihedral torsion* as “improper torsion”.

**Figure 1:**
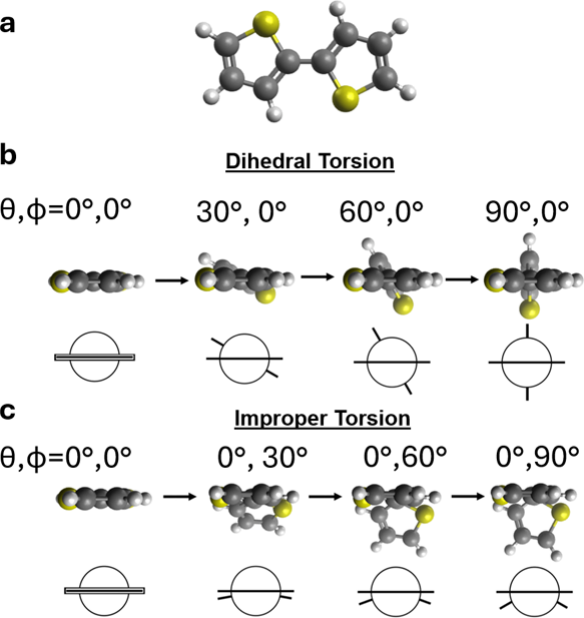
Improper vs proper intermonomer dihedral torsion in conjugated polymers. Conjugated polymers can experience two types of torsion: proper dihedral torsion and improper dihedral torsion. θ is hereon used to refer to the proper dihedral torsional angle, and ϕ to the improper dihedral torsional angle. (a) Model conjugated dimer (bithiophene) in a coplanar geometry. In (b) dihedral torsion, the twisting of monomers does not result in a change in the center of mass of the monomers. This is in contract to (c) improper torsion, in which an increase in the degree of bending accordingly shifts the center of mass of one monomer.

Both dihedral and improper torsion can break the planarity of conjugated polymer backbones, however, research has historically focused on dihedral torsion.^11,9^ Here, one widely applied technique technique for characterizing the drive toward coplanarity between adjacent monomers of a conjugated polymer is the torsional scan.^12–14^ In this method, the energy of a simple oligomer, such as a dimer, is calculated using quantum mechanics (QM) at planarity. Then, the dihedral angle between monomers is scanned at a set increment (often 10°) from planarity (0°) to 180 or 360°. At each step, the energy of the molecule is calculated. This progressive increase in intermonomer torsion interrupts electron delocalization across the backbone, and usually peaks when the monomers are perpendicular to each other at 90°. Interruption of delocalization is energetically unfavorable, and constitutes a significant component of the potential energy profile of a torsional scan.^5^ The resulting torsional energy profile over the intermonomer dihedral can be used to fit molecular mechanics (MM) force field (FF) parameters for large-scale molecular dynamics (MD) simulations. This profile defines the energy of deplanarization (i.e., how rigid the polymer is).

The torsional scan method characterizes the energy of one type of torsion, i.e., the conventional dihedral torsion.^10^ However, it is well-known that semiconducting polymers can also experience improper torsion between monomers (Figure 1). Like dihedral torsion, improper backbone torsion disrupts coplanarity and limits π-orbital overlap. Improper torsion and dihedral torsion can also occur simultaneously; given the dependence of electron delocalization on backbone geometry, it follows that these effects must influence the energetic barriers to both dihedral and improper torsion. This further implies an interdependence between the degree of improper bend and the energy of a given degree of dihedral torsion. However, even basic investigation of improper torsion at the QM level is beyond the coverage of current literature for organic semiconductors.^9^ As a result, the energy of dihedral torsion cannot be decoupled from the energy of improper torsion in a conventional torsional scan.

Recent advances in organic electronics have challenged conventional wisdom about torsional effects, that is, materials with large dihedral twists, which were expected to show low mobilities, have demonstrated excellent performance in some cases.^15,16^ These findings suggest that understanding of torsional effects is incomplete, especially with respect to the role of improper torsion. However, isolating the effects of improper torsion is challenging. Clashes that arise between atoms of the involved monomers lead to very large energy penalties, even at low degrees of improper torsion. In these cases, it can be hard to separate these nonbonded effects from the underlying covalent energies using conventional methods. An additional difficulty arises from the expected correlation between the energetics of improper torsion and those of dihedral torsion. For example, if electron delocalization is already partially blocked by an improper torsion, the barrier to additional dihedral torsion may be reduced. Due to this coupling, it is not possible to fully characterize the energetics stemming from improper torsion using a simple modification of angular or improper potentials. As a result, current models of improper torsion rely on terms taken from a general force field (i.e., OPLS, CHARMM, GAFF), and are focused on maintaining the rigidity of aromatic rings rather than considering intermonomer effects.^17–19^

Here, we present a comprehensive framework for modeling and separately quantifying the effects of proper and improper dihedral torsion in conjugated polymer repeat units. We applied this framework to a set of conjugated dimers and donor–acceptor units to characterize the relationship between electron delocalization and intermonomer backbone conformation. We used the electron delocalization data to create custom force field parameters that model the coupled improper–proper dihedral energy effects to evaluate potential accuracy gains in MM models of these materials. This approach yielded minor improvements in accuracy over existing methods. Additionally, we developed an interactive visualization tool that integrates molecular geometries, delocalized electron density distributions, and energy profiles. This tool is especially useful to new learners to facilitate their understanding of the relationship between conjugated polymer conformation and electronic structure. This work advances our fundamental understanding of conformational effects in conjugated polymers while providing practical tools for more accurate modeling of polymeric materials.

## Methods

### Isolating Delocalization Energy to Characterize Improper Potentials

To extract the energies arising from improper torsion between two aromatic monomer residues, we extended a previously reported methodology.^5^ This prior work introduced a method that uses a series of QM energy calculations to produce highly accurate parameters for proper dihedral backbone torsions. The torsional potential can be attributed to two primary components: (1) steric/nonbonded interactions and (2) covalent electronic effects. Since steric contributions, which often drive monomers away from planarity, are already accounted for in molecular mechanics models through nonbonded force field terms (van der Waals and electrostatic interactions), the methodology isolates these effects to prevent double-counting. This enables a potential solely associated with covalent delocalization interactions to be obtained, which was found to be strongly planarizing.^5^ Relying on QM calculations alone allows the planarizing energy of electron delocalization to be decoupled from steric interactions. This approach enabled accurate calculation the energy of dimer conformations with high degrees of steric clash. Such conformations pose a significant challenge to conventional methods, which rely on subtracting nonbonded energies calculated with a MM torsional scan from a QM torsional scan.^10^ We expand upon this method in the present work, where scans through a combined set of dihedral and improper angles are used to derive a coupled improper–proper (CIP) potential.

To isolate the energy related to nonbonded interactions, we used a three-step process (Figure 2). First, a set of torsional scans were conducted on an unmodified dimer starting from the coplanar structure. We generated a set of dimer structures with varying degrees of improper torsion, in increments of 5° from 0 to 30°, and in larger increments of 10° from 30 to 60°. These intervals were taken to reduce computational cost, and also because of the low likelihood of higher degrees of improper torsion (e.g., > 30°). We then performed a torsional scan of the intermonomer dihedral on each of these starting structures in increments of 10°, from 0 to 360°. For the second step, this process was repeated by conducting an identical set of scans with a hydrogenated dimer structure. This time, two hydrogen atoms were added to one of the monomers. For P3HT, the atoms were placed at the (2,5) positions. One hydrogen atom was added to the carbon atom across the ring from the intermonomer bond. The other was placed on the carbon atom that participates in the intermonomer bond, perpendicular to the aromatic ring in the direction of the ring’s normal vector (1.1 Å away). This unphysical hydrogen blocks electron delocalization, as well as any forces related to the energy of delocalization. For the third step, this process was repeated with another structural variant, in which one of the rings was replaced with a methyl group. We denote this structure a ”methylated monomer”. The hydrogen atoms in the methyl group were oriented in such a manner as to replace the atoms that would be bonded to the 2-carbon if a full ring was used. Likewise, the unphysical hydrogen atom was again placed in the same position as in the hydrogenated dimer (i.e., in an orientation that minimizes the energy) to minimize steric effects. Torsional scans of this methylated monomer were conducted as a function of improper angle (and fit to an OPLS-style dihedral form) to determine the energy associated with conjugation. The nonbonded energy (i.e., steric forces) was isolated by subtracting the energy associated with conjugation (i.e., determined using the methylated monomer) from the energy of the hydrogenated dimer. Likewise, the energy associated with electronic delocalization can be isolated by subtracting the calculated energy of the hydrogenated dimer from the energy of the simple dimer. A more detailed treatment of this protocol can be found in our previous work.^5^ After running the full set of scans for the given dimer, a unique set of OPLS-style dihedral parameters was fitted to the data produced by each of the torsional scans.^17^ This set of dihedral parameters was subsequently incorporated into a specialized force field using PLUMED to couple the improper and dihedral torsional angles.^20^

**Figure 2:**
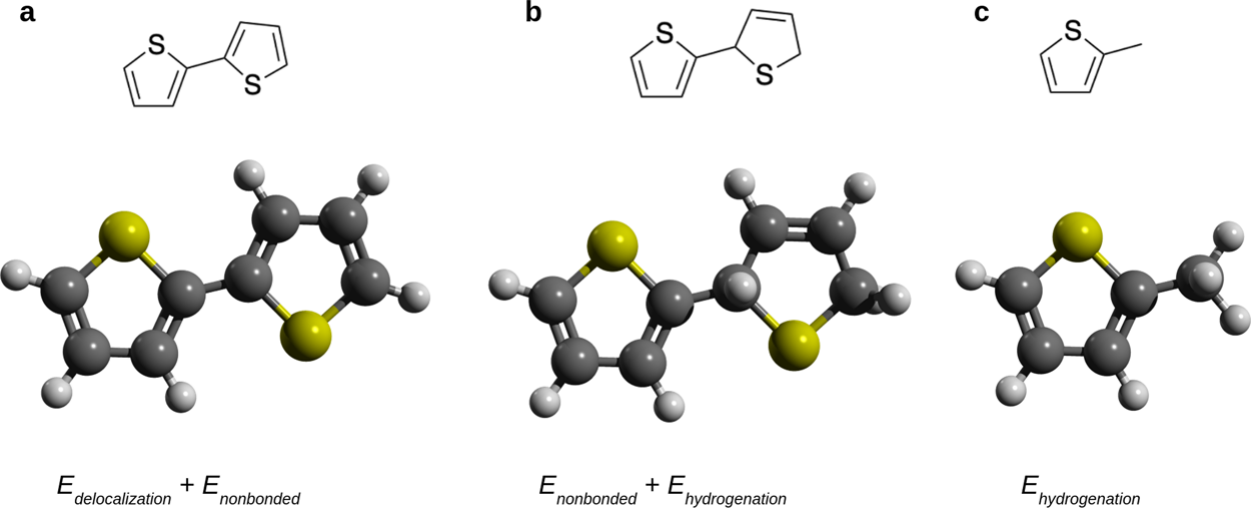
Method of isolating the energy associated with electronic delocalization. Torsional scans were conducted on (a) a standard dimer, (b) a hydrogenated dimer, and (c) a methylated monomer (i.e., where one monomer is replaced by a methyl group). By subtracting the calculated energy (relative to improper angle) of the hydrogenated dimer from the simple dimer, the delocalization energy can be isolated. Similarly, by subtracting the calculated energy of the methylated monomer from the hydrogenated dimer, the nonbonded energy can be isolated. Thus, this method allows one to decouple the energies related to electronic delocalization and steric forces.

### Selection of Materials

To determine the effectiveness of our method across a diverse array of π-conjugated polymers, we tested three representative polymers: poly­(3-alkylthiophene) (P3HT), polythieno­[3,4-*b*]-thiophene-*co*-benzodithiophene (PTB7), and poly­(naphthalene diimide) with a single bridging thiophene (PNDI-T). P3HT, a well-studied homopolymer, is often used as a model for semiconducting polymers.^21^ PTB7 was selected for its benzodithiophene residue, which is common in p-type donor–acceptor (D–A) polymers.^8^ Additionally, PTB7 offers the opportunity to investigate the effects of distinct regiochemical couplings within the monomer: an outward-pointing fluorine (“F-out” configuration) inward-oriented fluorine (“F-in” configuration). These configurations are treated separately in conventional torsional parametrizations and were also treated separately by us. Finally, PNDI-T was chosen because the naphthalene diimide backbone motif is common for n-type D–A polymers. In addition, PNDI-T has several characteristics that distinguish it from P3HT and PTB7. First, PNDI-T is highly sterically hindered in the planar state, causing it to adopt a twisted conformation.^13^ Second, the monomers that compose PNDI-T have much greater differences in electronegativity compared to PTB7. By including PNDI-T, we can examine how improper torsion affects aromatic residues with varying electronegativity differences—a key factor in tuning charge transport capabilities of D–A polymers. For computational efficiency and to focus on backbone conjugation, all polymers were modeled with methyl groups substituting the side-chains or R-groups.

### Error Quantification

To determine the relative accuracy of the method described above, we compare it to both a conventional parametrization (general OPLS force field parameters from LigParGen) and to our previously reported delocalization energy parametrization (where an isolated delocalization energy torsional profile was used to fit torsional parameters, but which did not account for improper torsions). We generated a randomly sampled set of dimer structures with varying improper and dihedral torsions, where all other internal degrees of freedom were restricted using the RIGID package in LAMMPS.^22^ OPLS FF parameters were applied to the dimers using LigParGen.^23^ All torsional parameters were set to zero and the simulation was performed at 800° K to allow the dimer to twist and bend freely, allowing the full conformational space to be sampled. Snapshots of random conformations were taken every 100 ps.

Quantum calculations were conducted for each conformation at the RI-MP2 level for a total of 100 conformations using QChem 5.4.^24^ These QM-calculated energies were used to compute the difference in energy between each sampled dimer conformation and the coplanar conformation. We then used MM parameters to facilitate comparison between: (1) a conventional dihedral parametrization (2) our previously reported delocalization energy-based MM parameters and (3) a new, specialized force field coupling dihedral and improper torsion. Using the QM-calculated energy differences as a reference, we calculated the error between the results from each of the above-mentioned MM methods with respect to the QM data. PLUMED was used to implement the specialized force field.^20^ The proper and improper intermonomer dihedrals were biased using PLUMED to apply our parameters, while the corresponding torsional terms were left unset in LAMMPS.

### MD Simulations of Polymer Films

To assess the effect of improper torsions in polymer films, we performed MD simulations using a standard version and our modified version of the OPLS FF for the three model conjugated polymers. For default FF, we used the standard OPLS-style force fields obtained from LigParGen.^25,26,23^ In the modified style, the parameters from LigParGen were used again, but the equilibrium intermonomer angles were changed. These angles were set to the optimal angle as determined by a geometry optimization using density functional theory - DFT. The optimization was performed at the BP86/def2-SVP level, shown by Matt et al. to produce accurate interplanar angles between conjugated moieties.^27^ Initial conformations of the polymer film were obtained from Packmol^28^ at a density of approximately $0.2\;\frac{g}{{\mathrm{cm}}^{3}}$ .

We then performed a quench-annealing MD protocol to equilibrate our films, using LAMMPS.^29^ The van der Waals and real space Coulomb cutoffs were set to 10 Å. A cubic spline was applied to the van der Waals to ensure smooth convergence and vanishing energies and forces at the cutoff (inner cutoff distance of 9 Å). The reciprocal space Coulomb interactions were computed with a particle–particle-particle-mesh solver, with an error tolerance of 10–6.^30^ Each MD simulation was initiated with 500 conjugated gradient steps, followed by gradual heating to 300 K over 0.5 ns (500,000 steps with an integration time step of 1 fs) dynamics in the canonical ensemble (*NVT* – constant number of particles *N*, volume *V* and temperature *T* = 300 K). A Nose-Hoover thermostat was used with a temperature relaxation window of 100 fs. This was followed by simulations in the isobaric, isothermal ensemble (i.e., constant number of particles, pressure and temperature, or *NPT*) for 5 ns at 400° K, before being cooled to 300° K over 0.5 ns. During NPT, we used the time-reversible measure-preserving Verlet integrators derived by Tuckerman et al.^31^ for the time integration. Finally, *NVT* production dynamics ran at 300° K for 1 ns, during which the improper angles were sampled.

### Interactive AICD Visualization

To make the communication of the quantitative results more visually intuitive, we developed an interactive web application to visualize the relationship between molecular structures and electron delocalization patterns. For each of the molecular conformers studied, we calculated the delocalized electron density using the “anisotropy of the induced current density” (AICD) method.^32^ This calculation required first identifying the π-bonding molecular orbitals (MOs) for each conformer. However, all of the conformers exhibited significant mixing between π-bonding and σ-bonding character in their MOs, making this process challenging (Figure 3a). To address this challenge, we developed a systematic approach to quantify the π-bonding character of each MO, in order to ultimately obtain the AICD isosurface for each conformer (Figure 3b). First, we performed a population analysis on each structure using Gaussian.^33^ We then analyzed the MOs using a scoring algorithm that evaluated two key criteria: (1) the alignment of orbital density relative to molecular bond vectors, and (2) the energy level of the orbital (Figure S1). The algorithm assigned higher scores to MOs with greater electron density parallel to bond vectors and less density directly overlapping these vectors; this approach builds on methods developed by Lu and Chen.^34^ MOs with energy eigenvalues greater than the population mean were also given higher scores. For each conformer, we selected the highest-scoring MOs for AICD analysis, which generated three-dimensional isosurfaces representing the delocalized electron density. All Gaussian calculations were performed using the Hartree–Fock method with a 6–31G* basis set, the defaults from the AICD input templates provided to us. Further exploration of the method and basis sets in AICD calculation is left to future study. These isosurfaces are interactively displayed over the molecular structures in our web application, allowing users to examine spatial relationships between molecular geometry and electron delocalization patterns (Figure 3c).

**Figure 3:**
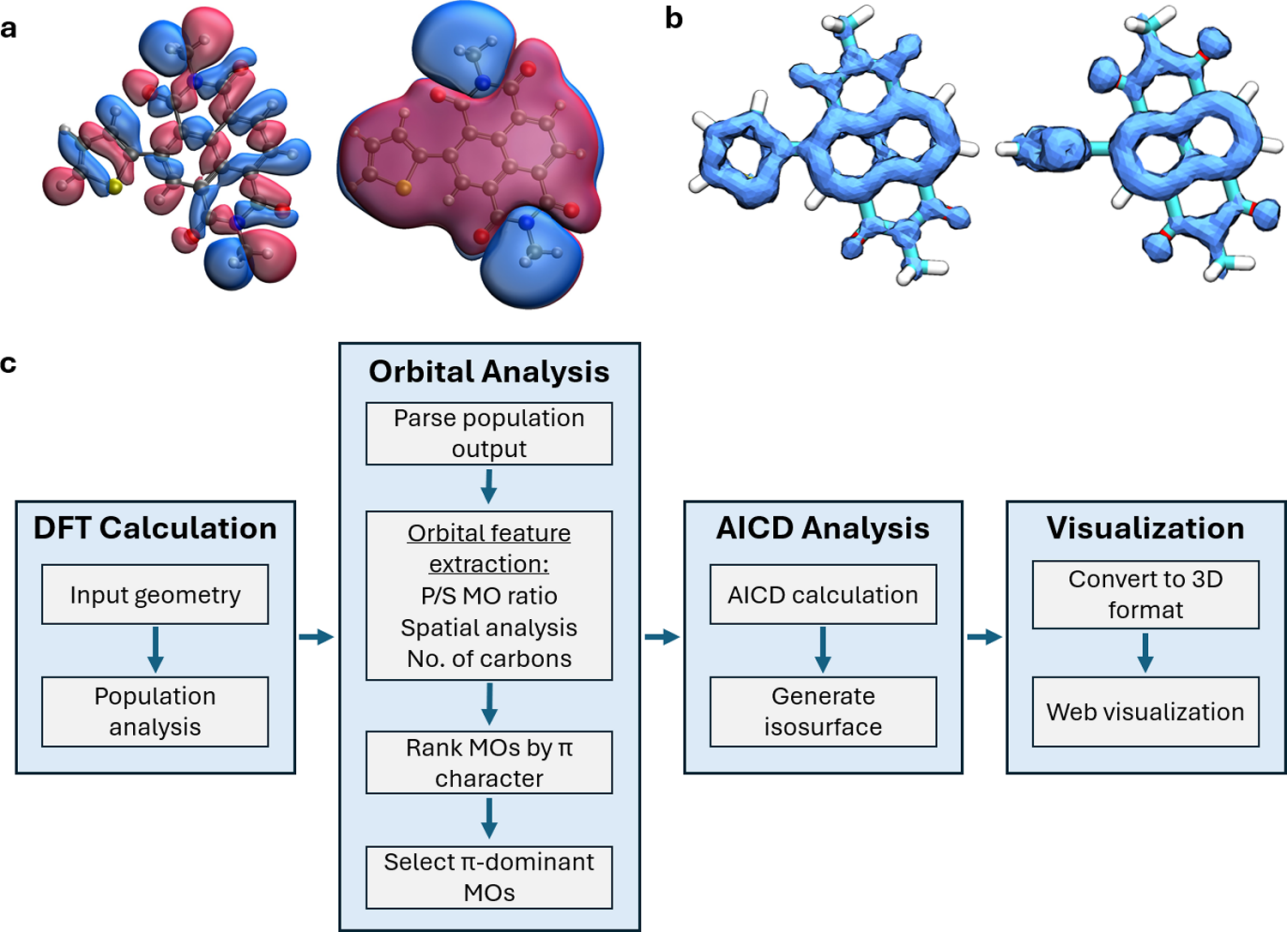
Motivation and protocol for obtaining AICD isosurfaces for conjugated polymers. (a) Shows examples of molecular orbitals of PNDI-T with low and high π-bonding characters. (b) Displays AICD isosurfaces of PNDI-T π-bonding MOs for 0° coplanar and 90° orthogonal conformations. (c) Describes the protocol used to generate the π-MO AICD isosurfaces for the conjugated dimers examined in this study.

## Results and Discussion

### Characterizing Presence of Improper Angles in Films

We determined the distribution of intermonomer improper angles for P3HT, PTB7, and PNDI-T using two angular parametrization methods (Figure 4). Surprisingly, the distributions of improper angles were statistically similar for all three polymers, across both angular parametrization methods. This gives rise to two important results. First, our simulations suggest that most conjugated polymer films will have some degree of improper torsion, regardless of how the intermonomer angles are parametrized. The means of the distributions were all around 4°, while the probability of observing improper torsional angle of 0° was very low (between 2.0 and 3.8%). Second, we found that significant improper angles occur in all conjugated polymers. The probability of observing large improper angles between monomers (i.e., 8° or greater) occur at least as often as the near-coplanar conformation (improper angle <2°), on average 15% vs 12% respectively. Thus, we conclude that improper torsion may be an important contributor to the overall energetics of conjugated polymer chains, and should be explicitly considered in force field parametrizations for systems where its energetic impact is determined to be significant. Moreover, any such consideration must include how improper and dihedral torsions are in fact coupled. We note that these simulations are performed at normal equilibrium conditions (i.e., at room temperature); elevated temperatures or nonequilibrium conditions could result in high degrees of polymer torsion, thus resulting in more frequent and larger improper torsion between monomers, as will be detailed presently.

**Figure 4:**
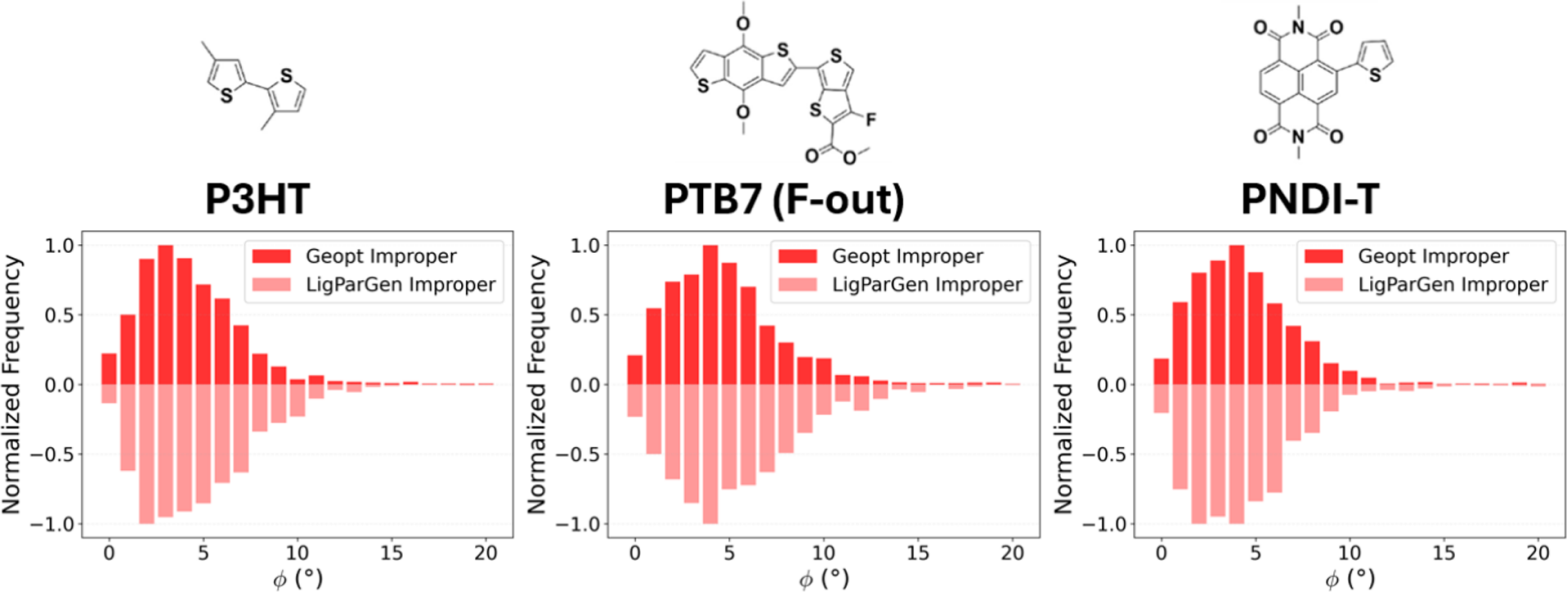
Comparison of improper angle populations in different angular parametrizations. Mirror histograms displaying improper angles of randomized polymer films with different angular configurations. In the LigParGen parametrization (light red, mirrored to negative values), the angular parameters were taken from an OPLS parametrization of the conjugated dimer. In the Geometry-Optimized parametrization (dark red, positive values), the angles were set to their equilibrium positions in a quantum mechanical geometry optimization. While geometry optimization was expected to lead to less improper torsion, both methods show modest amounts of improper torsion.

### Total Energy of Coupled Proper-Improper Torsion

When dihedral torsion (proper or improper) is induced on a dimer, the energy of the system changes relative to the torsional effect on nonbonded interactions and electronic delocalization. These nonbonded forces usually manifest as steric repulsion at the internuclear distances considered here. We first investigated the effect of coupled dihedral and improper torsion on P3HT, PTB7, and PNDI-T using conventional torsional scans (Figure 5). In these simulations, the energies associated with nonbonded interactions and electronic delocalization cannot be decoupled. We have previously found that at high degrees of dihedral torsion, steric clashes result in the nonbonded energy dominating over the delocalization energy.^5^ A similar case holds for improper torsion. For all polymers, overall energetic differences are dominated by a dramatic increase in steric energy at high degrees of improper torsion (Figure 5). Our simulations also indicate that certain combinations of dihedral and improper torsion can reduce the relative strength of steric clash compared to the 0° improper conformation. At large improper angles, the dihedral energies of all modeled polymers are skewed by the increase in the energetic penalty of nonbonded interactions. In P3HT, the energy of adjacent monomers increases sharply and decreases near 0° of dihedral torsion as the large sulfur atom becomes close to the hydrogen atoms in the adjacent ring. The F-in variation of PTB7 sees large variations in energy (both upward and downward) as the fluorine atoms approach the benzodithiophene monomer. Notably, these same variations in energies are not present in the F-out variation. For PNDI-T, there is a clear potential energy maximum (180°), a barrier over 3 times higher than the next largest local maximum. However, there is no clear energetic minima because all conformations suffer from steric hindrance.

**Figure 5:**
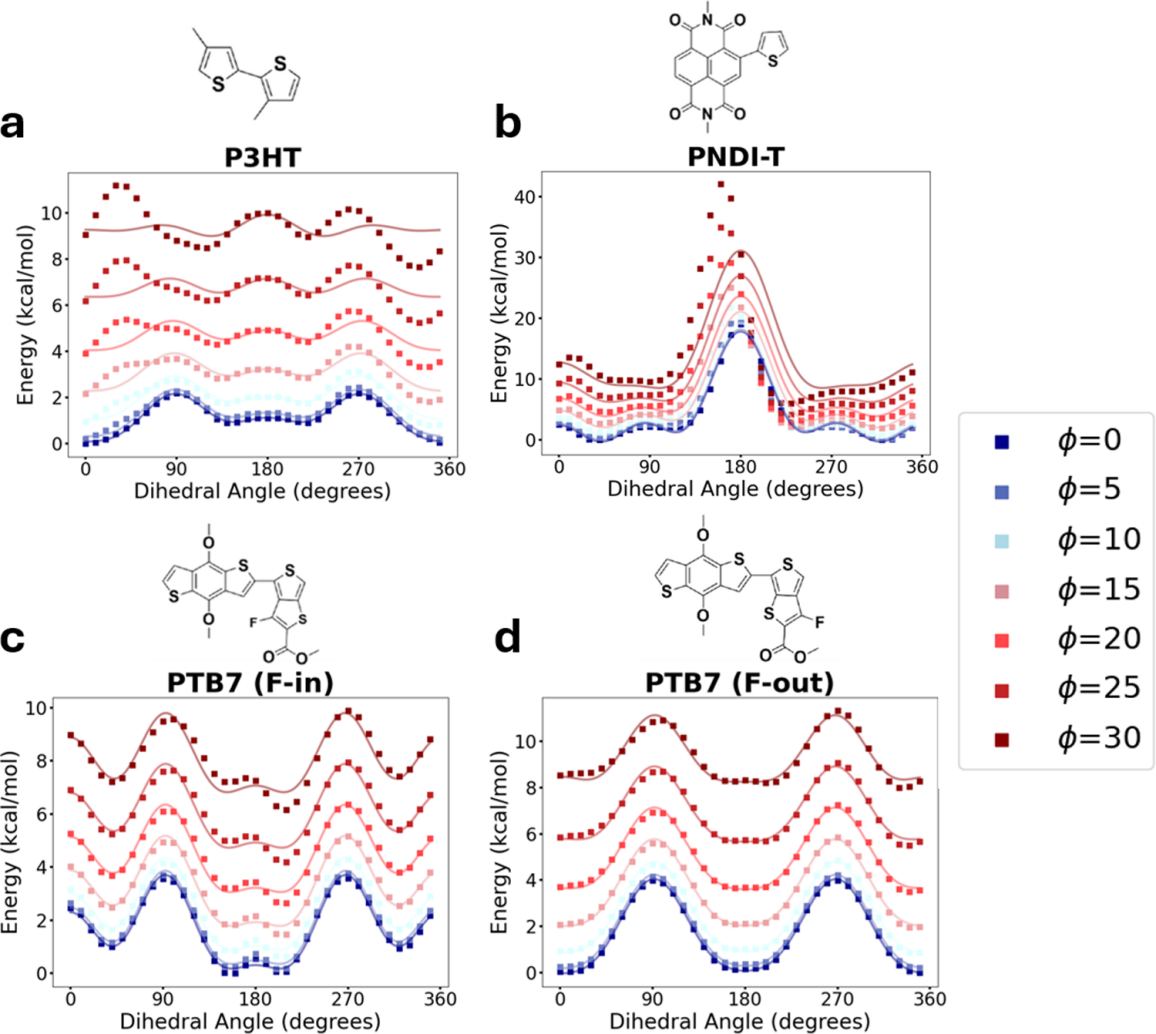
Total energy from QM proper-improper torsional scans. Energy calculated directly from QM torsional scans of adjacent monomers for (a) P3HT, (b) PNDIT, (c) PTB7 F-in and (d) PTB7 F-out relative to dihedral angle (from 0 to 360°) and improper angle (from 0° in blue to 30° in red). Scatter plot points are data, solid lines are OPLS dihedral fits.

These standard QM scans reveal that increased improper torsion significantly distorts the proper dihedral profile due to steric clashes between monomers. Since nonbonded interactions dominate these profiles, they cannot be directly used for parameter fitting. By isolating the delocalization energy (removing nonbonded components), we can successfully fit OPLS dihedral parameters, and understand how improper and dihedral torsion affect the drive toward coplanarity in conjugated polymers (Figure 6).

**Figure 6:**
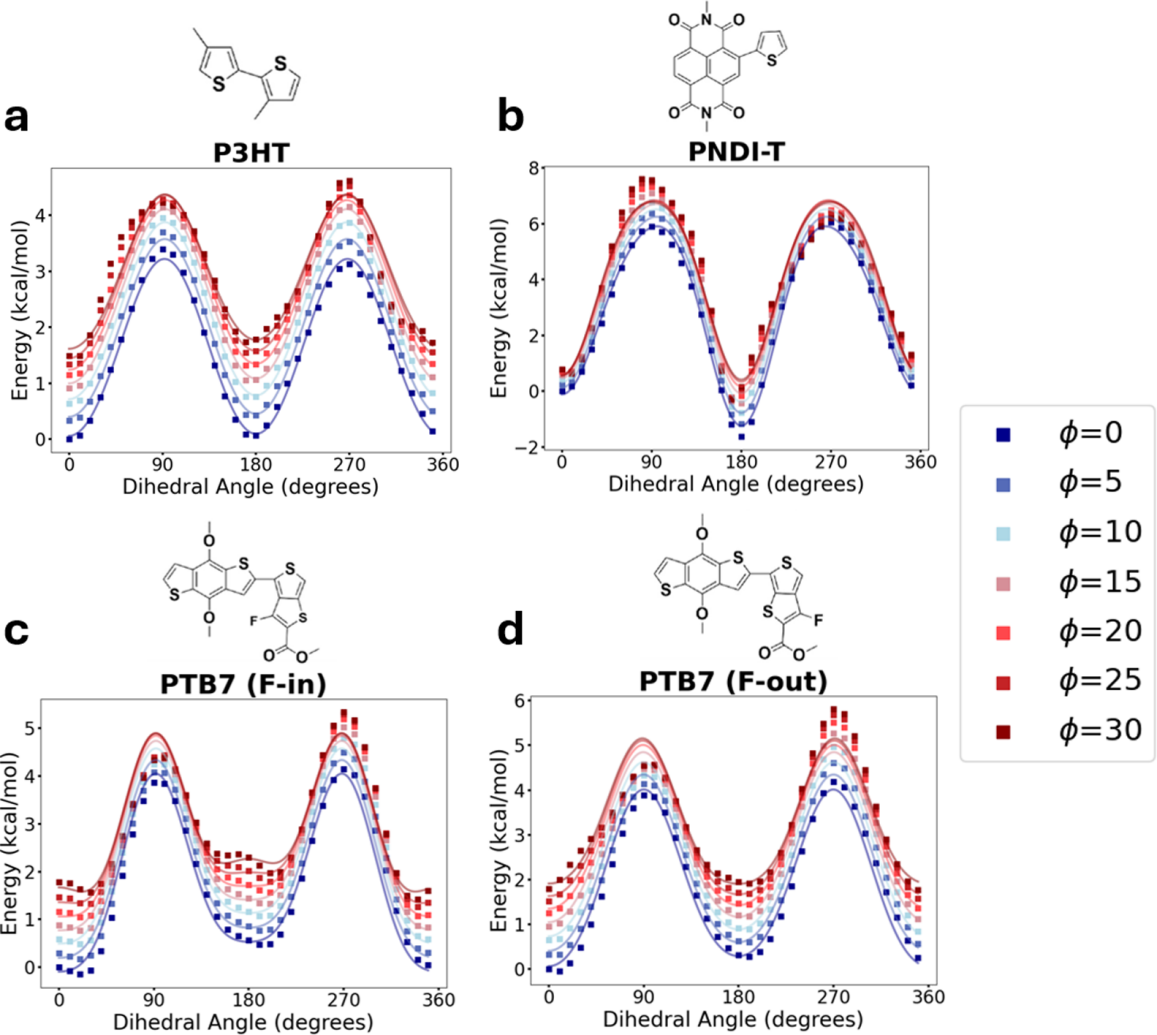
Isolated delocalization energy from QM for proper-improper torsional scans. Energy associated with electronic delocalization for (a) P3HT, (b) PNDIT, (c) PTB7 F-in and (d) PTB7 F-out relative to dihedral angle (from 0 to 360°) and improper angle (from 0° in blue to 30° in red). Scatter plot points are data, solid lines are OPLS dihedral fits. By removing the nonbonded energy (e.g., steric interactions) from each simulation, the effect of both dihedral torsion and improper torsion on the delocalization energetics can be elucidated.

### Delocalization Energy of Coupled Proper-Improper torsion

The delocalization energy profiles (without nonbonded forces) are similar for both PTB7 configurations (Figure 6), indicating that fluorine placement minimally impacts the intrinsic electronic preference for planarity. However, when nonbonded forces are included, the energy profiles differ substantially (Figure 5), revealing that steric effects from fluorine positioning—not changes in electronic delocalization—drive the observed conformational differences. The line shapes of the energy distributions for PTB7 F-out look similar, regardless of whether nonbonded energy is included or excluded in the calculation. This similarity suggests that the F-out conformation of PTB7 has the lowest degree of steric hindrance of the polymers considered here. Indeed, the line shape of the torsional profiles for P3HT, PTB7 (F-in), and PNDI-T changes significantly under increasing improper torsion, an effect that disappears after removing the nonbonded energy from the profiles.

Once the nonbonded energies are removed, it becomes evident that the potential barrier associated with dihedral torsion tends to decrease slightly as improper torsion increases in P3HT and in PTB7. That is, the total energy that can be lost or gained due to dihedral torsion is reduced as improper angle increases. Thus, increasing improper torsion impedes intermonomer conjugation, which reduces the total amount of energy associated with electronic delocalization available to be gained or lost. For all three polymers, there are energetic minima around dihedral angles near 0 and 180°, and maxima around 90 and 270°. For P3HT and PTB7, an increase in improper torsion decreases the height of the torsional barrier (the total amount of delocalization energy available to be gained or lost) by ∼0.33 kcal/mol for P3HT and ∼0.78 kcal/mol for PTB7. However, in PNDI-T, there is a slight increase in the torsional barrier of ∼0.27 kcal/mol (Figure 7a). This finding indicates that electron delocalization is not significantly obstructed, and may even be enhanced by improper torsion in this polymer, likely due to the large π-electron system of the naphthalene diimide monomer. This finding offers a suggestion as to why poly­(naphthalene diimide)-based polymers, which exhibit substantial degrees of backbone disorder, can maintain good electronic properties under large strains (e.g., in stretchable OFET devices).^35,36^

**Figure 7:**
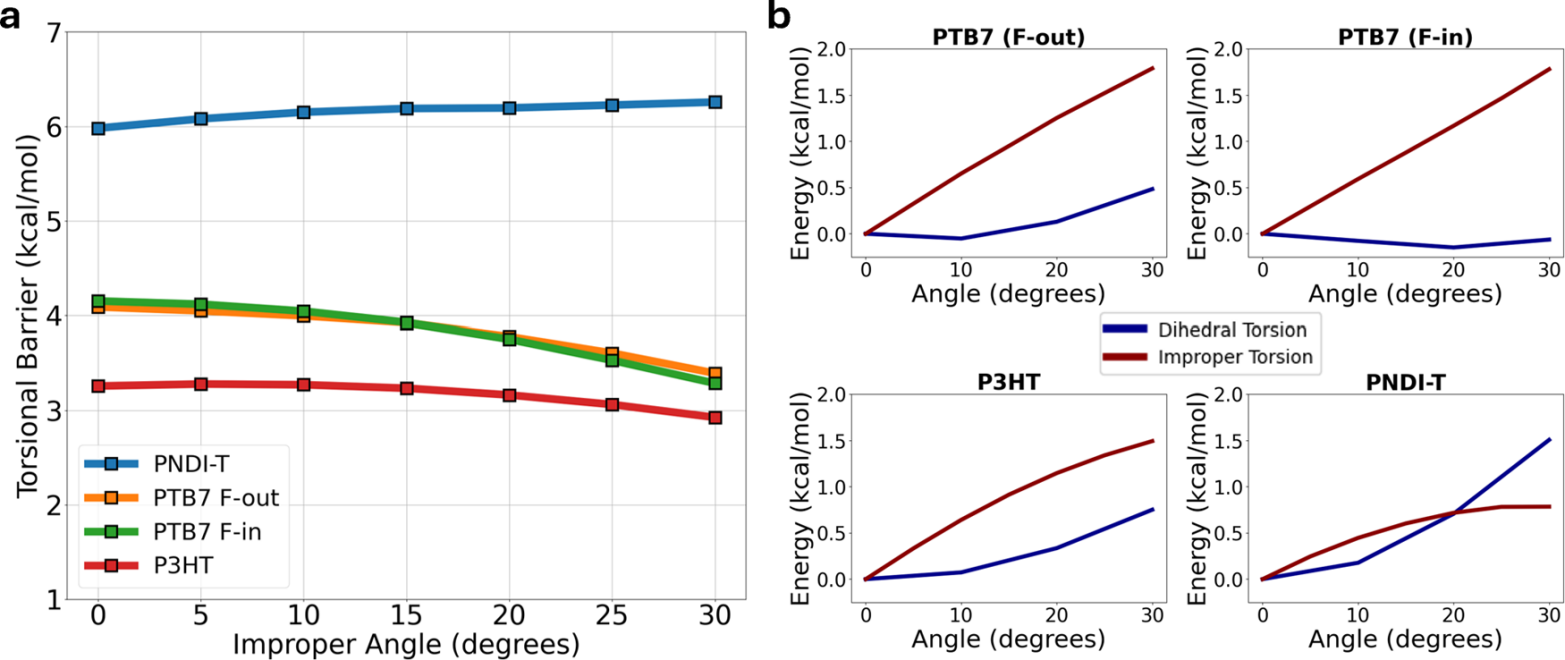
Delocalization energy barriers to proper and improper torsion. (a) Barriers to proper dihedral torsion as a function of improper angle for each of the polymers studied. The torsional barrier decreases slightly relative to increasing improper torsion for P3HT, PTB7 (F-in and F-out), but changes little for PNDI-T. (b) Energy with respect to isolated dihedral torsion and improper torsion for each of the polymers studied. For P3HT and both PTB7 variants, increasing the improper angle has a much higher energetic cost compared to dihedral torsion. This does not hold true for PNDI-T, which possesses a much larger conjugated structure compared to the other polymers.

We further quantify this effect by calculating the dihedral torsion barrier, taken as the difference in delocalization energy between the first minimum and the average of the two maxima from the dihedral scan, for each polymer (Figure 7a). We find that for P3HT and PTB7, the barrier to dihedral torsion decreases slightly as the improper angle increases. In these cases, intermonomer delocalization is disrupted by the onset of improper torsion, so the impact of dihedral torsion on the delocalization energy is attenuated. This is supported by the large barriers to improper torsion in these molecules, which are more than double the respective barrier to dihedral torsion at low angles (Figure 7b). In these cases, more pathways for electronic delocalization (i.e., conjugation) are broken than are created.

Conversely, PNDI-T shows a comparatively small resistance to improper torsion. The barrier to dihedral torsion in PNDI-T shows a corresponding insensitivity to the improper angle. In fact, PNDI-T shows a very small increase to the barrier to dihedral torsion with increasing improper angle. This suggests that appreciable conjugation can still be maintained, and possibly even increased, in PNDI-T despite modest degrees of improper torsion. This result is interesting because all of the polymer systems considered here are predicted to have non-negligible improper torsion (Figure 4). Our simulations of PNDI-T suggest that favorable delocalization energetics can be maintained despite a disordered backbone. Further study is needed to determine whether this amenability to improper torsion is related to the high degree of push–pull effect in PNDI-based polymers. (For example, from the greater differences in electron-withdrawing character between the donor and acceptor moieties). Likewise, future work is needed to determine if and when improper torsion makes more conjugation than it breaks. In other words, if and when improper torsion allows for additional overlap within the π-system, thus maintaining conjugation (i.e., pathways for charge carriers) over larger length scales. Our simulations suggest that understanding the effect of improper torsion on electronic delocalization can guide the rational design of D–A polymers that have high charge transport properties under high strain. That is, whether favorable electronic properties can be maintained in a conjugated polymer thin film (e.g., by additional overlap of π orbitals) when strain induces or increases torsion.

### Evaluating Accuracy

We obtained energies for a range of dimer conformations using the coupled proper-improper parametrization method and calculated the errors with respect to energies obtained from QM. We compared these errors to those of two reference models to assess the impact of the new parametrization method on the accuracy of the molecular mechanics model (Table 1). For comparison, we used a standard OPLS parametrization as well as our previously reported delocalization energy parametrization.^5^ The improved parametrization enhanced accuracy compared to conventional force fields across all studied polymers. Table 1 shows the mean error reductions achieved using our coupled improper-dihedral parametrization compared to two other existing FFs. Error is defined as the difference between energy obtained by a molecular mechanics model and the reference QM model (aug-cc-PVTZ RI-MP2) for a given conformation. Mean errors are taken across a comprehensive set of randomly sampled conformers, as described in the “Error Quantification” subsection. We compare the mean error of our improper-inclusive FF to the mean error of the standard OPLS FF and to that of our previously published “OPLS-DE” delocalization energy FF.

**Table 1:** Mean Error Reductions

polymer	mean error change vs conventional parametrization (kcal/mol)	mean error change vs delocalization parametrization (kcal/mol)
P3HT	–0.54	–0.23
PTB7 F-out	–0.11	–0.03
PTB7 F-in	–0.62	–0.29
PNDI-T	–1.12	–0.04

The improvements were modest in comparison to our previous OPLS-DE parametrization method. This suggests that while incorporating coupling effects between proper and improper dihedral torsions in conjugated polymer models does increase accuracy, the majority of improvements over conventional models stem from better parametrization of proper dihedral torsions (as implemented in OPLS-DE). The limited additional gains from considering proper-improper torsional coupling indicate that the OPLS-DE method already captures most of the achievable accuracy improvements in these systems. For oligomers and polymers longer than the dimers studied herein, larger π-electron systems with long-range delocalization may increase the strength of the driving effects captured by this method. Additionally, despite the relatively small absolute differences to the torsional barriers, these modes may store substantial energy across an entire polymer chain, possibly driving changes in the thermal and mechanical behavior of the polymer model. The potential of this method to improve model accuracy with respect to long-range delocalization and thermomechanical behavior of conjugated polymers is an important topic that is left open for future study.

### Developing an Interactive Visualization for π-Electron Delocalization

After developing the computational framework necessary to quantitatively investigate the relationship between electron delocalization and backbone conformation, we identified a gap in our ability to effectively visualize and communicate these results. Existing visual representations of π molecular orbital systems proved difficult to translate into an intuitive understanding of the complex relationship between electronic structure and molecular geometry, particularly when conveying these results to researchers outside the field of molecular simulations. To address this limitation and enhance the broader impact of our work, we developed an interactive web application, shown in Figure 8a. This app was designed to facilitate exploration of electron delocalization in conjugated polymers. Initially, we attempted to visualize delocalization using the highest occupied molecular orbital (HOMO); however, this approach was ineffective for many structures, particularly nonplanar systems. The HOMO electron density in such cases appeared asymmetrical and did not provide a clear visual representation of delocalized electrons. The challenge of visually representing electron is well-documented in the literature, where several alternative methods, including natural bond orbital (NBO) analysis,^37^ electrostatic potential (ESP) mapping, and electron localization function (ELF) calculations,^38^ have been proposed to visualize electron delocalization.^39–42^ However, most of these techniques are optimized for planar, aromatic systems and have limited applicability to nonaromatic or nonplanar conjugated polymers.^32,34^

**Figure 8:**
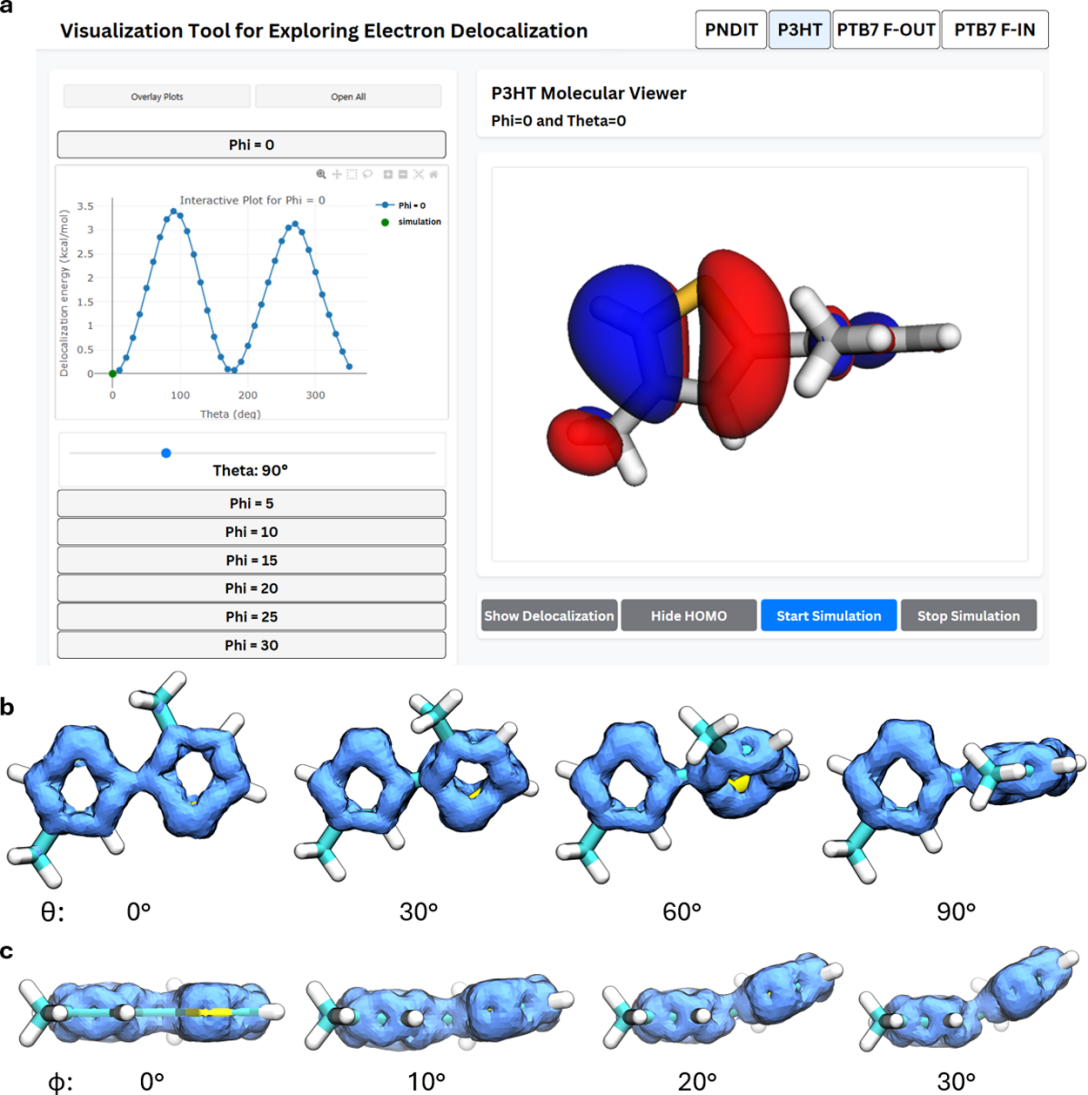
Interactive web application design and delocalization isosurface examples (a) An interactive web application for exploring the 3D structure of conjugated dimer conformers, their delocalization energy, HOMO density plots, and associated delocalized electron density maps. Delocalized electron density (AICD) isosurfaces can be loaded and rendered in our web application, and are shown for our model of P3HT at progressive degrees of (b) proper dihedral torsion and (c) improper dihedral torsion.

To overcome this challenge, we identified the anisotropic induced current density (AICD) method as a promising alternative for visualizing π-electron delocalization in conjugated polymers.^32^ The advantage of AICD lies in its ability to capture current density distributions induced by external magnetic fields that are independent from the underlying electron density. AICD isosurfaces thus provide an intuitive representation of electron delocalization beyond conventional depictions. However, applying AICD to conjugated polymers required the development of a systematic method for selecting appropriate molecular orbitals from population analysis. As described in the “Interactive AICD Visualization” section, we established a robust selection process that enables automatic identification of the relevant π molecular orbitals. This automation allows us to efficiently generate isolated AICD isosurfaces for π molecular orbitals across large data sets. Additionally, we implemented a conversion method to render these representations in an interactive 3D format, enhancing interpretability beyond the program’s default output of 2D static images. These developments enabled us to integrate the AICD visualizations as our delocalization isosurfaces in the web application. The resulting visualizations not only improve our understanding of these systems but also serve as a valuable tool for broader dissemination of insights into conjugated polymer behavior.

## Conclusions

This work sought to understand the effect of improper torsion on the energetics of three model polymers: P3HT, PTB7, and PNDI-T. Our simulations suggest that varying degrees of improper torsion would be common in the polymer chains that compose a solid sample. This finding is significant because of how improper torsion affects conjugation along a polymer chain. Improper torsion decreases electron delocalization when planarity is broken. Second, improper torsion increases electronic delocalization if additional overlapping of the π-orbitals is induced. In general, we find that the first effect is stronger than the second, and thus an increase in improper torsion generally results in a greater energetic barrier to torsion for all polymers here except PNDI-T. As a result, improper torsion is generally an obstacle for electronic delocalization. Additionally, we find that energetic penalties from steric interactions obscure the energetic penalties associated with electronic delocalization relative to increasing improper torsion. By modifying our computational method to isolate the energy related to electronic delocalization, we show that the barrier to torsion changes as improper angle increases. Similarly, by isolating the effects of both styles of torsion, we determine that improper torsion generally imposes a stronger energetic penalty compared to dihedral torsion. However, the delicate balance between delocalization and steric interactions with varying molecular conformation results in some exceptions. In particular, simulations of PNDI-T suggest that it is possible for adjacent monomers in a conjugated backbone to be highly twisted and still maintain conjugation. This finding suggests an explanation as to why some D–A polymers can maintain good charge transport properties while highly strained (e.g., in stretchable organic transistors). Finally, we show that a computational method that considers both improper and dihedral torsion can be used to simulate all three model polymers more accurately, although the increase in accuracy is modest.

Our findings indicate that the inclusion of a modified characterization of improper torsion helps better describe the energetics of conjugated polymers. Thus, consideration of the improper torsion within a conjugated system is recommended for highly accurate models of semiconducting polymers. This importance stems from the effect of improper torsion on both the overlap of π-orbitals as well as internal disruption of π-systems. In addition, improper torsion can directly affect the energetics that drive proper torsion. Improper torsion is even more so likely to be important when high degrees of torsion are present in general (e.g., films under strain, polymers deposited from melt, films annealed at high temperatures). These results will be of interest to researchers interested in molecular systems that experience high steric pressures, particularly with regard to spectral and thermomechanical property calculations.

While description of improper torsion improves the accuracy of modeling conjugated polymers, further steps should be taken to quantify the effects of improper torsion. Judicious exploration of the placement of additional hydrogen atoms to optimally block electron delocalization and isolate steric hindrance would be preferred, as high steric hindrance can still cause difficulties in modeling. For example, we show that the behavior of PNDI-T differs greatly from both P3HT and PTB7 when improper torsion is introduced between two monomers. Further exploration of highly sterically hindered polymers (particularly D–A polymers with asymmetric monomers) can provide information about the disruption of electronic delocalization in conjugated polymers. For example, our findings suggest that PNDI-T can maintain favorable electronic delocalization at high degrees of improper torsion. Therefore, further investigation of improper torsion in D–A polymers elucidate the energetic effects of π-orbital overlap. Additional work in this area could offer significant insights into the high mobilities of modern D–A polymers, many of which are amorphous or take advantage of short-range aggregation. Thus, accurate understanding of how chemical structure affects the morphology (as mediated by chain conformation and packing structure) of a polymer solid offers an avenue for improving predictions of its physical (e.g., electronic and mechanical) properties. In doing so, computational simulations of conjugated polymers can be used to facilitate the rational design of new polymeric materials.

## Supplementary Material



## Data Availability

The data from QM calculations for this paper are available on Zenodo. DOI: 10.5281/zenodo.15284080 The Zenodo data set will continue to be updated with additional simulation data as it becomes available. Code availability: the code used to generate the input files in order to perform the scans and fits described in this work is available at https://github.com/atlas-nano/aromodel and will continue to be updated to improve usability and extensibility. Web application: A live version of the web application is currently hosted at https://chemcompute.org/simulations/submit/Energy+Displacement+of+Electron+Delocalization.

## Notes

Detailed overview of process for identifying π-dominant MOs from Gaussian population analysis (PDF)
