# Supplementary material for: Quantifying the Effect of Intermonomer Improper Angles on Electron Delocalization in Conjugated Polymers
Source: J Phys Chem B. 2025 Jul 14;129(29):7642–53. doi: 10.1021/acs.jpcb.5c02849 (PMC12302062; doi:10.1021/acs.jpcb.5c02849)
Supplement: Supplementary file 1 [file jp5c02849_si_001.pdf]

Supplementary Information for

Quantifying the Effect of Intermonomer Improper Angles on Electron Delocalization in Conjugated Polymers

Robert S. Ramji, Andrew T. Kleinschmidt, Shruti Bhamidipati, Leon Zhang, Alexander X. Chen, Tod A. Pascal,* Darren J. Lipomi*

Aiiso Yufeng Li Family Department of Chemical and Nano Engineering, University of California, San Diego - 9500 Gilman Drive, Mail Code 0448, La Jolla, CA 92093-0448, USA

Department of Chemical Engineering, University of Rochester
4510 Wegmans Hall, P.O. 270166, Rochester, NY 14627-0166, USA

*Authors to whom correspondence should be addressed: tpascal@eng.ucsd.edu, darren.lipomi@rochester.edu

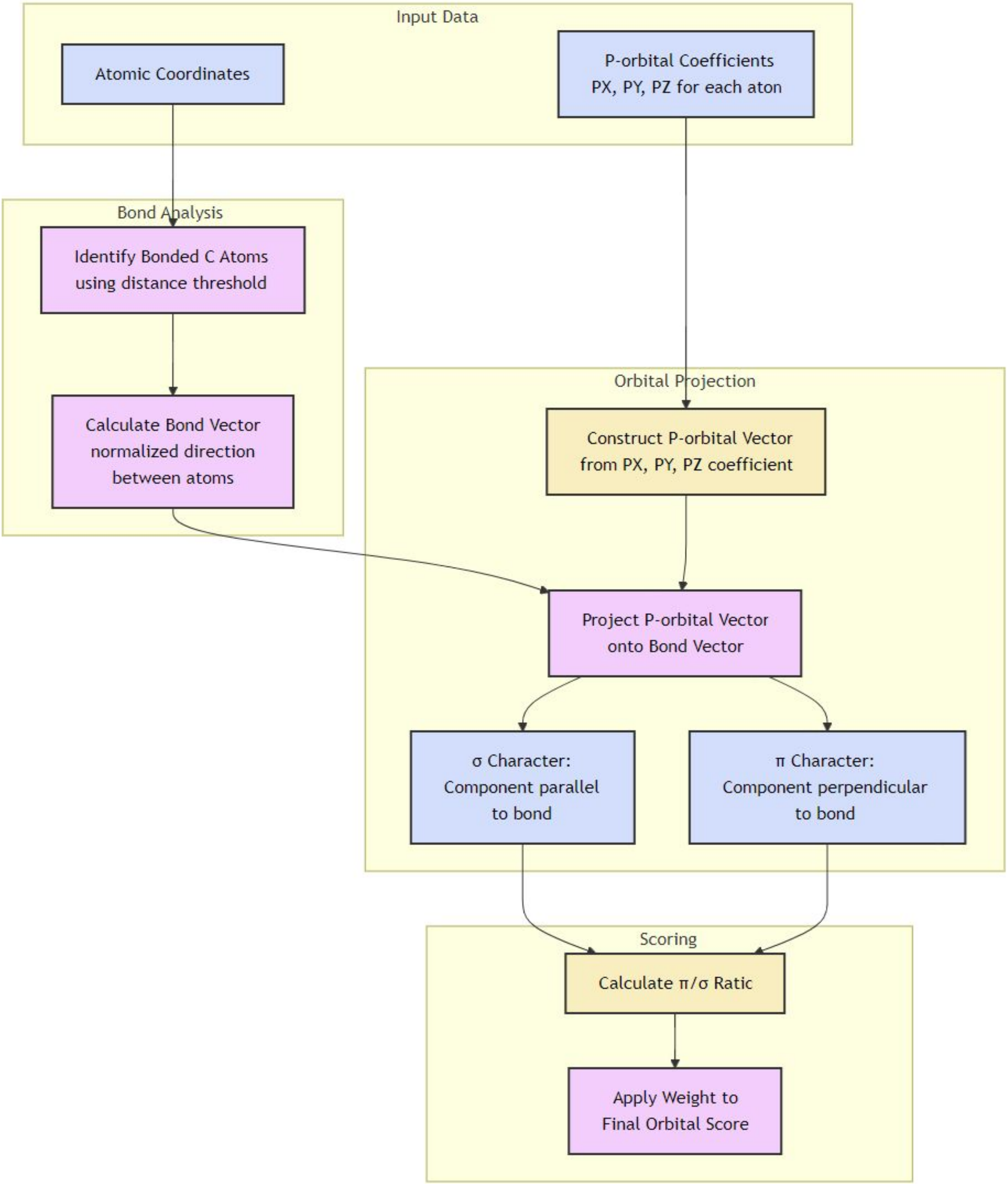

Figure S1. Detailed overview of process for identifying π-dominant MOs from Gaussian population analysis data prior to computing AICD isosurfaces. This step is necessary to reduce σ-bonding contributions to the AICD isosurfaces in order to more clearly visualize the relationship between intermonomer geometry and π orbital delocalization.
